# Mendel’s laws, Mendelian randomization and causal inference in observational data: substantive and nomenclatural issues

**DOI:** 10.1007/s10654-020-00622-7

**Published:** 2020-03-23

**Authors:** George Davey Smith, Michael V. Holmes, Neil M. Davies, Shah Ebrahim

**Affiliations:** 1 MRC Integrative Epidemiology Unit (IEU), Bristol Medical School, University of Bristol, Oakfield House, Oakfield Grove, Bristol, BS8 2BN UK; 2 Medical Research Council Population Health Research Unit (MRC PHRU), Department of Population Health, University of Oxford, Nuffield, Oxford, UK; 3 Clinical Trial Service Unit and Epidemiological Studies Unit (CTSU), Nuffield Department of Population Health, University of Oxford, Oxford, UK; 4 London School of Hygiene and Tropical Medicine, London, UK

**Keywords:** Mendelian randomization, Nutritional epidemiology, Causal inference, Alcohol, Genetic epidemiology

## Abstract

We respond to criticisms of Mendelian randomization (MR) by Mukamal, Stampfer and Rimm (MSR). MSR consider that MR is receiving too much attention and should be renamed. We explain how MR links to Mendel’s laws, the origin of the name and our lack of concern regarding nomenclature. We address MSR’s substantive points regarding MR of alcohol and cardiovascular disease, an issue on which they dispute the MR findings. We demonstrate that their strictures with respect to population stratification, confounding, weak instrument bias, pleiotropy and confounding have been addressed, and summarise how the field has advanced in relation to the issues they raise. We agree with MSR that “the hard problem of conducting high-quality, reproducible epidemiology” should be addressed by epidemiologists. However we see more evidence of confrontation of this issue within MR, as opposed to conventional observational epidemiology, within which the same methods that have demonstrably failed in the past are simply rolled out into new areas, leaving their previous failures unexamined.

## Introduction

Reading what Mukamal, Stampfer and Rimm (henceforth MSR) consider to be a “review” [1] of Mendelian randomization (MR) we felt we had been transported back many years. Their essay will read oddly to anyone acquainted with MR, as it mainly recapitulates limitations to the approach discussed in the first extended exposition [2]. These have stimulated the development of a wide range of sensitivity analyses, of which MSR appear unaware. Indeed, MSR fail to reference a single paper on MR methodology. Rather than use up pages of the *EJE* outlining the basics of MR—of which most readers will likely be aware—we refer MSR to a few of the many contemporary actual reviews [3–6]. Instead we address the substantive issues we can extract from their essay. These are (1) MR is receiving more attention than the general concept of causality, and has been too eagerly adopted; (2) the name “Mendelian randomization” contributes to its inappropriate popularity and should be changed; (3) MSR provide critical commentary on MR of alcohol and cardiovascular disease (CVD), an issue in which their conventional observational studies have produced widely promoted findings; (4) MSR suggest that MR “should be treated with the circumspection that should accompany all forms of observational epidemiology”; with the latter we largely agree.

## Mendelian randomization: Is it too popular?

**Fig. 1:**
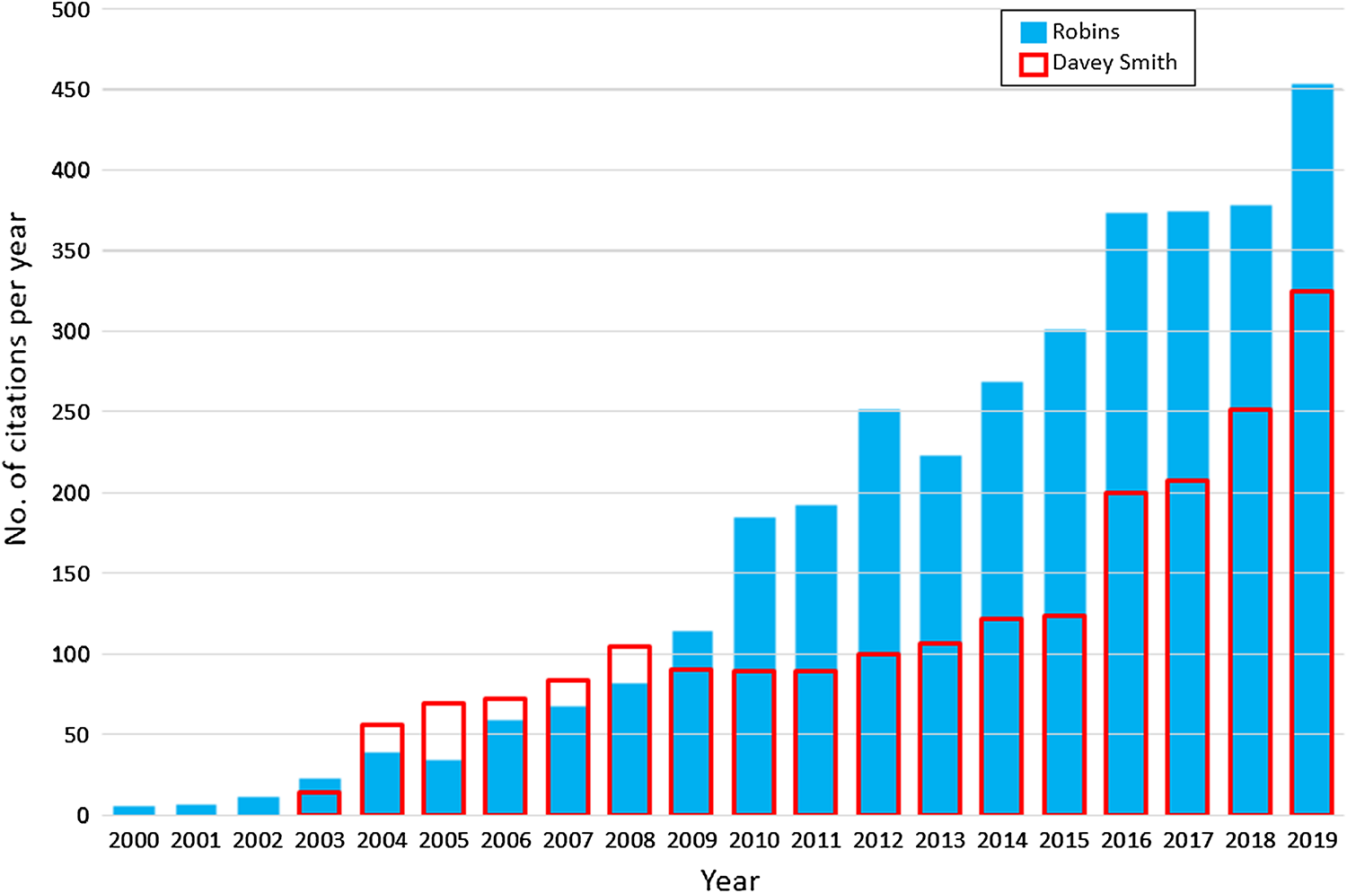
Google Scholar citations to the papers that heralded a marked upturn in use of the terms “causal inference” [8] and “Mendelian randomization” [2] within epidemiology

MSR present a figure from Google searches which purports to show that “worldwide interest in MR has steadily increased over the last 10 years, while that of causality more generally has not” [1]. We were surprised by this claim, as we had investigated the increased interest in causal inference in epidemiology recently [7] and were not reassured by attempting to replicate MSR’s search strategy. The issue of relevance to readers of the *EJE* will relate to causal inference and MR in the epidemiological field. We therefore examined citations for the inflexion-point papers for (“causal inference” and epidemiology) [8] and (“Mendelian randomization” and epidemiology) [2]. These are the most highly cited papers in their class and ones that heralded a rapid growth in publications in their respective areas. As Fig. 1 shows there has been interest in both.

**Fig. 2:**
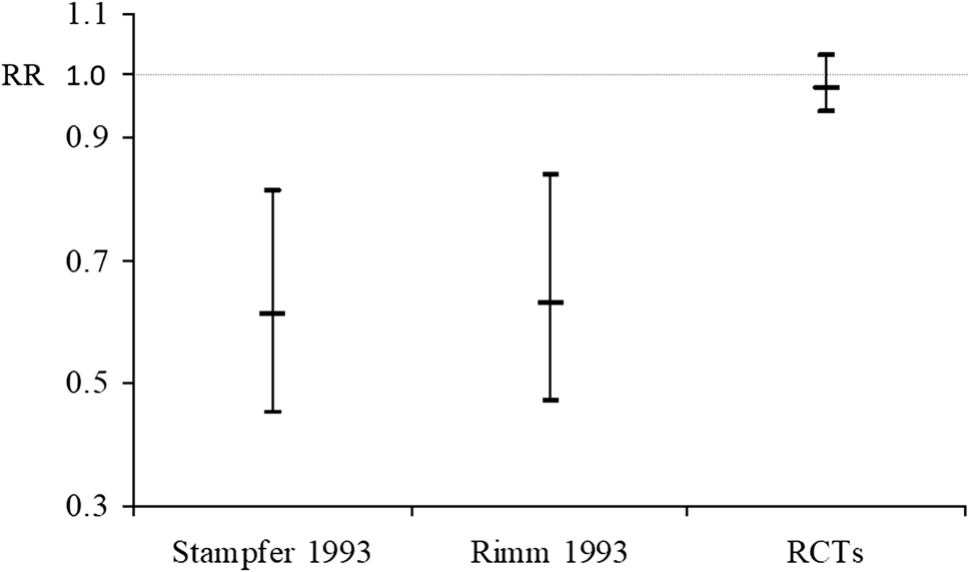
Exemplar of misleading epidemiological research, used to illustrate the need to develop alternatives to naïve observational analyses, in an early review of MR (reproduced from [24]). Vitamin E supplement use and risk of CHD in two observational studies [9, 10] and in a meta-analysis of RCTs [111]. RR: relative risk

What requires no demonstration is that the proportion of publications within epidemiology that are concerned with causal inference in general, or with MR in particular, have increased. We intuit that epidemiological interest in both has been driven by the high-profile failures of conventional observational epidemiological research of the sort carried out by MSR—such as on vitamin E supplement use [9, 10] or hormone replacement therapy (HRT) [11] and coronary heart disease (CHD), among other topics. Randomised controlled trials (RCTs) failed to corroborate their epidemiological findings. These examples of MSR and their colleagues’ work featured prominently in critiques of epidemiology from different perspectives appearing around the millennium [12–15]. RCTs testing hypotheses they advanced (e.g. [16, 17]) have continued to produce null results since then (e.g. [18, 19]). Concern with the high profile failure of these conventional epidemiological studies was a major stimulus for formulating ways of strengthening casual inference within epidemiology, including the introduction of MR [2, 20–23]. Indeed, MSR made substantial contributions to the development of MR, by repeatedly producing exemplars of epidemiological studies which failed to reliably identify efficacious targets for interventions to improve population health. Illustrating their contribution, Fig. 2 is reproduced unchanged from an early paper on MR [24], where it was used to encapsulate the failures of conventional epidemiological studies, making crystal clear the need for new methods.

MSR consider the popularity of MR disproportionate. Consideration of academic attention to the vitamin E supplementation and HRT papers by MSR [9–11] casts doubt on this; they have each been cited more than any empirical MR paper. Other highly cited contributions by MSR—e.g. suggesting that alcohol [25–27], folate supplementation [28], vitamin C supplements [29] and vitamin D [17] protect against CVD, that selenium protects against prostate cancer [16], etc., illustrate that high citations are not an indicator of scientific validity. In comparison the most highly cited empirical MR paper is one demonstrating that the inverse association between circulating HDL cholesterol (HDL-C) and CHD is unlikely to be causal [30], as has now been shown by many RCTs [31]. Whilst the first MR study on HDL-C appeared before the first large HDL-C raising trial was reported [32], the highly cited MR paper was a latter collaborative analysis across a large number of studies [30]. In their work, by contrast, MSR have simply assumed that HDL-C protects against CHD, seeing it as mediating substantial components of the apparently beneficial effects of alcohol and HRT on CHD [25, 26, 33–35].

We agree that with the advent of two-sample MR, and the ease of carrying out these analyses, MR studies can be performed with too little thought. Indeed we have published a critique of this practice [36], which has some similarities with the rapid rise in publication of meta-analyses [37].

## Mendelian randomization: What’s in a name?

MSR do not like the name MR, suggesting instead that it be referred to as genetic instrumental variables analysis. They appear to consider this a novel suggestion, although those acquainted with the MR literature will know this proposal has been advanced several times before, most coherently in 2008 [38], to which we responded at the time [39].

MSR dislike use of the term “randomization” in MR for two reasons. One is that it assumes that “alleles distribute freely within open populations; in essence, the latter assumes that one’s parents are also randomly assigned” [1]. The first extended exposition of MR [2] introduced the concept within the framework of parent-offspring studies, which do not require this assumption:

Mendelian randomization is most clearly seen in parent–offspring designs that study the way phenotype and alleles co-segregate during transmission from parents to offspring. In matings in which at least one parent is heterozygous at a polymorphic locus, the frequency with which one of the two alleles from a heterozygous parent is transmitted to an offspring with a particular disease or phenotypic characteristic can be evaluated. If there is no association between allelic form and the disease or phenotypic characteristic, each of the two alleles from the heterozygous parent has a 50% probability of being transmitted to the offspring [2].

The analogy with an RCT was introduced with respect to this design:

A shift from this 50/50 ratio indicates an association between disease or phenotypic characteristic and the alleles at this locus. This study design is closely analogous to that of RCTs as by Mendelian principles there should be an equal probability of either allele being randomly transmitted to the offspring. [2]

In 2003 it was not possible to utilise this approach:

Such studies may be difficult to carry out however, both because of problems in obtaining data from parents and offspring (particularly when parents may be dead) and because they generally have lower statistical power than case-control studies carried out within whole populations, rather than within families. [2]

Given the lack of adequately powered studies utilising a parent-offspring design, population data—and even these were sparse—could be used, but the MR was only approximate:

Of course populations share much common ancestry and the genetic make-up of individuals can be traced back through the random segregation of alleles during a sequence of matings, but associating genetic markers with disease risk or phenotype within such populations is not as well protected against potential distorting factors as are parent–offspring comparisons. Thus the Mendelian randomization in genetic association studies is approximate, rather than absolute. [2]

MSR further complain that the word “randomization” in MR assumes “meiosis randomly assorts maternal and paternal chromosomes into individual gametocytes” [1]. They clearly dispute RA Fisher’s observation that:

Genetics is indeed in a peculiarly favoured condition in that Providence has shielded the geneticist from many of the difficulties of a reliably controlled comparison. The different genotypes possible from the same mating have been beautifully randomized by the meiotic process…..Generally speaking the geneticist, even if he foolishly wanted to, could not introduce systematic errors into the comparison of genotypes, because for most of the relevant time he has not yet recognized them. [40]

RA Fisher, seen as the instigator of RCTs, developed these by analogy to what is now called Mendelian randomization [41]:

A connection between our two subjects which seem not to be altogether accidental, namely that the “factorial” method of experimentation, now of lively concern so far afield as the psychologists, or the industrial chemists, derives its structure and its name, from the simultaneous inheritance of Mendelian factors. Geneticists certainly need not feel that the intellectual debt is all on one side [40].

Perhaps MSR have more information on the prevalence of transmission ratio distortion—which would be required for the non-randomization (beyond, of course, variants in close proximity on the same chromosome to those being transmitted, as discussed in [2])—than is available to us. Our review of this issue failed to yield such evidence [42]; luckily definitive studies will now be possible, as large genotyped parent-offspring studies are becoming available.

Regarding naming of the approach, frankly, we don’t care. There have been a wealth of proposals from 2004, when Tobin et al. suggested “Mendelian deconfounding” [43] until 2020, when MSR unconsciously reiterate earlier suggestions, but now with the catchy acronym GIVA [1]. Sadly, the acronym GIV (genetic instrumental variables) has been adopted by a causal inference method that uses genome-wide data [44], so MSR will need to come up with another name and acronym. The label Mendelian randomization was only adopted because one of us had chanced upon the term—used for an ingenious design to utilize the HLA compatibility of siblings of children with acute myeloid leukaemia to evaluate the effects of bone marrow transplantation [45]—and appropriated it when proposing use of molecular genetic data to evaluate the potential causality of homocysteine on CVD [46]. Our lack of attachment to the name is public[47], with the suggestion that, given a second chance, the formally correct “human reverse genetics” might have been a good—but less catchy—choice. If MSR want to call it GIVA that’s fine, although they should perhaps acknowledge that there are forms of inference that can come from MR that are not encapsulated by conventional understanding of instrumental variables analysis [2, 39, 48].

## A twice-told tale: Does alcohol reduce cardiovascular risk?

**Fig. 3:**
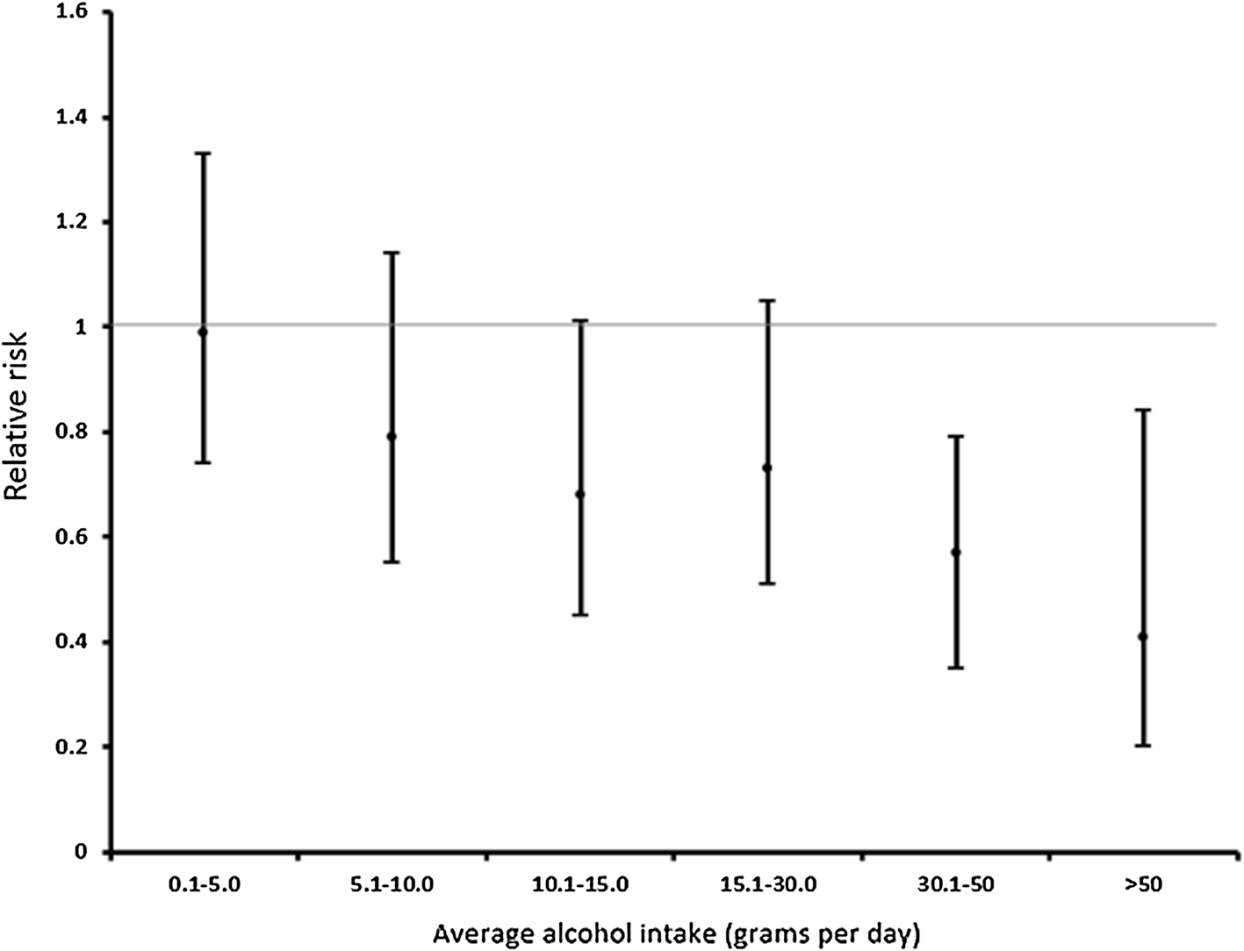
Relative risk of coronary heart disease by daily alcohol consumption, compared to non-drinkers. Data from Rimm et al. [25]

MSR frame their discussion of potential biases in MR analyses in relation to epidemiological evidence on alcohol and CVD. Their work has, over many decades, promoted cardiovascular benefits of alcohol consumption. For example, Rimm and Stampfer (as first and senior author) stated in a 1991 paper—using essentially the same methods they have continued with up to this day—that their findings “support the hypothesis that the inverse relation between alcohol and risk of CHD is causal” and that 40% of the benefit was mediated by increased HDL-C [25]. Their findings suggested a linear decrease in CHD risk with increasing alcohol consumption, with the lowest rate being in the highest category, those drinking at least the equivalent of 3 US pints of the then prevailing beer a day (Fig. 3). In countless further papers the basic story—a protective effect of alcohol mediated though HDL-C (with fibrinogen being brought in as an additional mediator [26, 27])—has been reiterated, with the extension of the apparent protective effects of alcohol to hypertensive men [49] and diabetic women [50]. It is therefore not surprising that MSR do not like the MR findings suggesting that alcohol increases blood pressure and the risk of total CVD [51–53].

MSR’s critique of MR therefore focuses only on issues that they perceive as invalidating MR studies of alcohol and CVD. There are many more potential limitations that those interested in more in-depth analysis can consider [2, 4, 5, 54]. Here we’ll comment under MSR’s headings on the issues they raise.

### Confounding

MR studies can suffer from confounding because ancestry can influence both the distribution of genotypes and outcomes in study populations. Obviously, ancestry cannot bias genotype-outcome associations between siblings, which is why MR was hypothetically introduced in a within-family context [2]. Now there are large sibling-pair datasets that allow these studies to be performed [42, 55], and for established biomedical relationships they produce the expected results.

With the huge sample sizes now available in studies such as UK Biobank, even a small influence of residual population stratification can confound genotype-phenotype associations and bias MR estimates, as we have demonstrated [56, 57]. Additionally study sampling can generate collider bias [58], as there is automatic conditioning on criteria for study inclusion, which will include willingness to participate and survival up to participation date. Collider bias generates associations between variables in a sample that can be biased downwards or upwards from the associations within the source population. We have demonstrated genetic influences on participation [59, 60], and have begun outlining options for recovering valid estimates in such situations [61].

It should be remembered that conventional observational studies are also susceptible to bias by ancestry, it has just hardly been considered, beyond adjusting or stratifying for self-reported ethnicity. In MR studies genome wide data are used in addition to self-reports, which allow finer adjustment. Further, we have not seen the collider bias introduced in sample selection—e.g. of health professionals who are willing to participate in a study—considered in reports of such studies. Ironically we discussed an example of where collider bias was likely to have generated a spurious “protective” effect of alcohol on stroke in an early MR paper [62].

MSR suggest ancestry might have biased findings in a meta-analytic MR study of a polymorphism in the alcohol metabolizing ADH1B gene [63]. The subpopulation they consider is not of large enough proportion of the total sample to produce substantial bias.

The influence of parental genotypes on offspring through the environment provided—stretching from the intrauterine period through postnatal life—has been studied in genetic epidemiology [64, 65] and referred to as “dynastic effects” in MR contexts [42]. Dynastic effects can be investigated though use of parental genotype or parental non-transmitted alleles conditional on offspring genotype [66]. Indeed, applying this MR approach suggests that maternal alcohol consumption leads to lower offspring educational attainment [67], which could explain the weak ADH1B-educational attainment association that MSR comment on. Regarding genetic variants MSR state that MR “must consider the actual origin of their presence in an individual’s genome—the genome of one’s biological parents” [1]. We agree, which is why we introduced MR in the context of parent-offspring studies [2], have elaborated on this approach now it is feasible [42], and are demonstrating that, as consideration of biological and social realities would lead one to expect, for many disease processes such biases in MR are not seen, although for social processes—such as educational attainment—they are evident [55]. We have also utilized MR to demonstrate assortative mating by alcohol consumption and thus by ADH1B genotype [68], which may account for this variant being out of Hardy-Weinberg equilibrium in some investigations.

A major form of confounding in naïve observational studies is that ill-health leads to a reduction or cessation of alcohol consumption, often many years before the serious disease events or death that are the study outcomes. Illness does not influence genotype, which is why MR can provide powerful insights into situations in which reverse causality is clearly problematic—such as observations that higher BMI apparently protects against lung and other cancers, which is not supported by MR [69].

### Weak instrument bias

MSR discuss weak instrument bias, but not in a way that would be recognisable to those familiar with instrumental variables analysis. Weak instrument bias is generated by statistical weakness of association between the genetic instrument and exposure of interest, and in single sample MR (instrument-exposure and instrument-outcome association both from the same study) this biases findings to the (confounded) observational exposure-outcome association; however in two sample MR (instrument-exposure and instrument-outcome from non-overlapping samples) it biases findings to the null [70]. Conventionally if the instrument-exposure F statistic is > 10 then there is likely to be little to no weak instrument bias [71]. In most MR studies of ADH1B and (particularly) ALDH2 the F statistics are so far above 10 that this is a non-issue [72].

A problem not part of weak instrument bias, although MSR consider it is, is exposure misclassification. Simple observational studies are more susceptible to this than MR studies; the use of genotypic averages (often based on very large samples per genotype) considerably lessens the impact of reporting bias by individuals. If an exposure is measured with error, its association with an outcome will generally under-estimate the causal effect of the exposure on the outcome. In contrast, as is well known, instrumental variable estimators are unbiased if the exposure is non-differentially measured with error [73].

### Pleiotropy

**Fig. 4:**
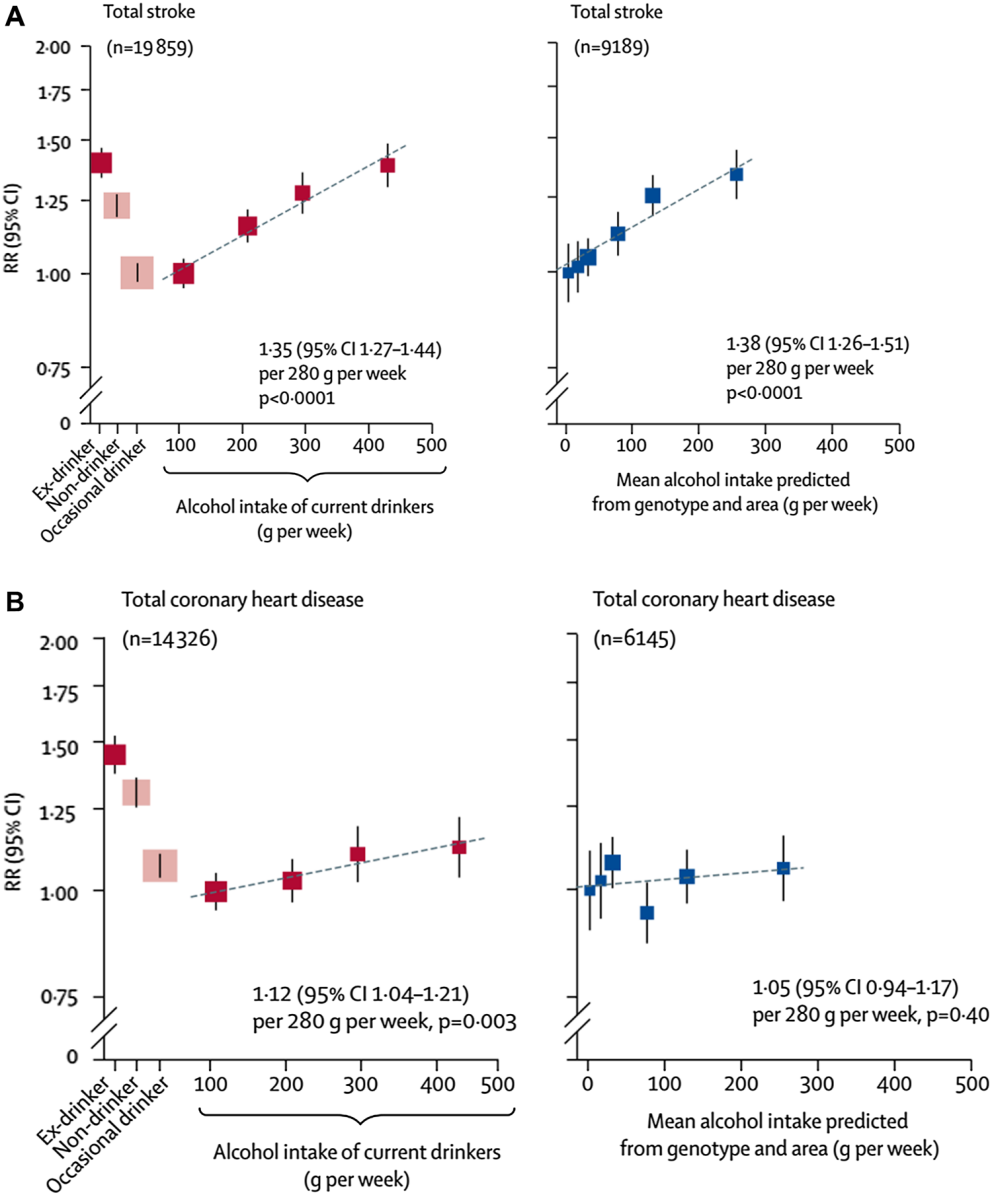
**A** Relative risk (RR) of stroke according to drinking patterns (left hand panel) and MR analyses (right hand panel) in men. **B** RR of coronary heart disease according to drinking patterns (left hand panel) and MR analyses (right hand panel) in men [51]

Horizontal pleiotropy [54] which, in our example, would involve a genetic variant influencing alcohol consumption and an outcome by independent pathways, is a major concern for MR [74]. Vertical pleiotropy, in which a genetic variant influences multiple traits because it has a primary influence on alcohol, which in turn influences downstream traits, is what MR depends upon [54]. Model organism studies suggest the latter is a more common phenomenon than the former [75, 76]. There is an extensive arsenal of sensitivity analyses that allow for valid estimation in the presence of horizontal pleiotropy [42, 74]. In the case of alcohol the most credible is utilising a group in which genotype does not associate with exposure. In studies in some East Asian locations women hardly drink alcohol. The ALDH2 rs671 null variant is prevalent in these populations, leading to marked symptoms amongst alcohol drinkers. In men polymorphism in rs671 relates very strongly to alcohol consumption. In women alcohol consumption is very low independent of genotype. In men the variant will convey the effects of both alcohol and any horizontally pleiotropic pathways; in women, only the pleiotropic effects will be seen. In men but not women the variant relates strongly to lower blood pressure and lower HDL cholesterol; among women these associations are not seen [52, 53]. This provides convincing evidence that alcohol elevates blood pressure and HDL-C. Furthermore, since sex is clearly not influenced by ALDH2 genotype, stratifying on sex is not stratifying on a collider [58]. Region of residence will also not be materially influenced by ALDH2 genotype, and thus joint stratification by sex and region will create groups in which the variant relates to a very widely differing extent to alcohol consumption. Horizontally pleiotropic effects of the variant will not differ between groups. Thus the manner in which the ALDH2-alcohol association across groups scales up to the adverse CVD consequences of alcohol consumption provides powerful evidence on the dose-response causal effects of alcohol [51]. The difference between the naïve observational association of alcohol with stroke (which shows the usual J-shaped curve generated by confounding and reverse causation, as seen in most observational studies) and the dose-response increase in stroke seen in the MR analyses is stark (Fig. 4a). For CHD the contrast is between the markedly J-shaped observational association and a null estimate from MR (Fig. 4b).

MSR comment on acetaldehyde as potentially distorting MR results on CVD for the variant in ADH1B. The disease specific toxicity of acetaldehyde can be easily identified using MR principles. Alcohol consumption among men is very low in ALDH2 homozygous null variant carriers (HNV), is intermediate in heterozygotes (HET) and is about twice the heterozygote level in homozygous functional allele carriers (HFA) [52]. Levels of alcohol-produced acetaldehyde is lowest in the HNV group, highest in the HET group (who drink alcohol but do not clear the acetaldehyde efficiently) and intermediate in the HFA group. For conditions influenced by acetaldehyde—oesophageal and head/neck cancer—the highest rates of disease are seen in the HET group and the lowest in the HNV group [77, 78]. The higher disease risk in HET than HNV demonstrate the contribution of acetaldehyde to carcinogenesis; the higher risk in HFA than HNV demonstrate that alcohol consumption is a major driver of disease incidence[77, 78]. If acetaldehyde were influencing CVD or its risk factors then the same shape of relationship between genotype and the outcomes would be seen, which they are not[51, 52]. The level of acetaldehyde generated by alcohol in ADH1B variant carriers is very considerably smaller than is seen with ALDH2 genotype. The notion that the small difference in acetaldehyde with ADH1B has “pleiotropic” effects on CVD and its risk factors that are not seen with the order of magnitude greater difference in acetaldehyde with ALDH2 is simply incoherent.

Regarding drinking behaviours (binge drinking and units) these are problematic to investigate in conventional observational studies—given confounding, misreporting and reverse causation—unless one believes that, as Rimm’s data suggest (Fig. 3), the highest level of drinking is the best strategy for cardiovascular health. As we have seen MR can produce estimates of the shape of the relationship between alcohol and health outcomes, and Rimm’s apparently linear decrease in CHD risk up to the heaviest drinkers could be tested thought the application of non-linear MR [79]. MSR suggest that MR studies showing “lower risk of cardiovascular disease associated with variants linked to lower alcohol consumption may well be proving the harm of binge drinking” [1]. However, across the sex and residential area groups there will not be a perfect scaling of effects of genotype on mean alcohol consumption and the proportion of binge drinkers. Indeed across the lower consumption groups the proportion of binge drinkers will be so low as to not credibly produce the outcomes seen. The linear effect across mean alcohol categories for stroke events in Fig. 4a is also seen for mean blood pressure, HDL-cholesterol and ɣ-glutamyltransferase [51]; these differences could not be produced by the plausible proportion of binge drinkers in these groups. This illustrates the essential nature of epidemiology as a science of group-level outcomes [80], with aggregation at the level of genotype reducing bias from measurement and reporting error, together with removing the under-estimated effects of reverse causality. In principle the issue of the effect of different components of drinking behaviour could be investigated through using multivariable MR [81], with genetic variants robustly related to any of reported binge drinking, total alcohol intake and a composite risk-taking behaviour measure all being utilised. Whether this will be possible is speculative at this time, but it could allow some interrogation of the issue.

MSR’s suggestion that the absence of an ADH1B effect on HDL-C is due to weak instrument bias is not plausible, as discussed above. It could be due to pleiotropy or the variant being in linkage disequilibrium (LD) with another variant that influences HDL-C. In MR there are many sensitivity analyses that utilise multiple genetic variants related to a trait [74]. As we have seen, the more powerful ALDH2 variant, which also allows stratification to exclude bias due to pleiotropy or LD—clearly demonstrates an effect of alcohol on HDL-C [51, 53]. Furthermore, using multiple variants in largely European-origin populations with a sample of ~ 1M, a clear effect of alcohol on HDL-C is seen, which is robust to stringent interrogation with sensitivity analyses [82]. As HDL-C does not influence CVD risk [31, 32] any failure of ADH1B to recapitulate the effects of alcohol on HDL-C will not distort the estimates of alcohol on cardiovascular outcomes. It may, however, herald other potential biases, which is why the use of multiple instruments and a wide range of sensitivity analyses not dependent on a single instrument are routinely instigated in contemporary MR studies.

### Adaptation

MSR consider MR “owes its popularity due to its ostensible ability to test exposures of interest in adulthood” [1], but MR is being increasingly used to investigate the effects of intrauterine exposures (using maternal non-transmitted alleles) [66] and has been widely applied to many childhood outcomes. MSR go on to suggest that MR cannot address issues of exposure changes over life, but consider Rimm’s (Fig. 3) demonstration of the linear decrease in CHD with increasingly heavy drinking. The zero or very limited drinking categories could contain many “young and adolescent binge drinkers” who quit due to adverse consequences of alcohol, on which the investigators probably have no reliable data, and thus have findings biased by this. If data on earlier life drinking were available on a sufficiently large sample then MR could be carried out within the different reported categories of earlier drinking behaviour, with less confounding and bias than in conventional observational epidemiology. A detailed consideration of the meaning of MR estimates with respect to interventions at different stages of life and with different durations is available [4], and MR can provide reliable separation of some exposures acting at different periods of life, such as childhood and adulthood adiposity [83].

## How to be circumspect

MSR state that MR “must be treated with all of the circumspection that should accompany all forms of observational epidemiology” [1]. We agree, and wonder how such circumspection applies to the myriad statements by MSR on the causal nature of the cardiovascular protection from alcohol, e.g. “the evidence indicates that the association between moderate alcohol consumption and lower risk of CHD is causal and that abstaining from alcohol could be considered a risk factor for CHD” [84]. MSR have also repeatedly made strong causal claims regarding raised circulating HDL-C mediating a substantial proportion of the apparently protective effects of alcohol, but HDL-C has been robustly demonstrated to have no protective effects in many large RCTs and MR studies [30–32]. Fibrinogen, the second most important mediator according to MSR, appears non-causal in MR studies [85]—and we suspect given this evidence there will never be an RCT targeting fibrinogen. A valid causal claim by MSR is that “half of the beneficial effect of moderate alcohol intake is due to increased HDL-C concentrations” [27]. This is probably true: best estimates suggest elevated HDL-C produces zero benefit, and doubling this equals zero. We would support an RCT of long-term difference in alcohol intake, but following cancellation of the proposed NIAAA MACH15 trial [86] for well documented reasons [87–89] it is unlikely this will ever happen.

MSR view investigations of folate supplementation and CVD as an appropriate use of MR [1]. Their discussion implies that conventional observational, MR and RCT results agree, but this is not the case. MSR’s influential early papers on the apparent effect of plasma homocysteine and folate intake—which reduces homocysteine—on CHD[28, 90] suggested a dose-response effect of folate in north American populations. They concluded that their “results suggest that any widespread increase in folate intake will have a favourable impact on CHD rates” [28]. Both MR [91, 92] and many RCTs [93] in such populations show this is spurious. MSR now consider folate supplementation reduces stroke in low folate intake populations. We are not convinced by even this conclusion, but what is clear is that their original observational findings regarding CHD were spurious. MSR’s conventional observational studies made other strong claims—for example, that folate would reduce blood pressure [94]—that we note they do not revisit. Reading the current RCT and MR evidence on this issue may explain why.

MSR discuss two applied MR issues: alcohol and folate. We think their disappointment regarding MR could be an abreaction to their earlier enthusiasm for the incorporation of genetic variants in epidemiological studies to strengthen causal inference. In a paper concluding that their genetic analysis provided support for the notion that alcohol was protective against CHD, partially through HDL-C, MRS presented a prototype of MR reasoning:

Some have suggested that the inverse association between moderate alcohol intake and the risk of myocardial infarction does not represent a true causal relation, but rather that alcohol is a surrogate for favorable socioeconomic or lifestyle factors associated with a reduction in risk. It is unlikely that the ADH3 genotype is associated with these potentially confounding factors, and we observed no such association in our data. [95].

They considered that:

Associations observed in nonrandomized epidemiologic studies may be attributed to potentially confounding factors. Observed associations between the risk of a disease and the presence of functional variants in genes that lead to the metabolism or transduction of the factor that underlies the disease add substantial support to the idea that the exposure to the factor is directly related to causation. [95].

In a statement we endorse, they opined that:

Improving our ability to identify specific lifestyle and environmental factors as causes of a given disease may prove to be one of the main benefits of the study of common variants in metabolic genes and disease. [95].

We approvingly cited this paper in our initial exposition of MR [2], although given the small study sample and the growing evidence of publication bias in the genetic epidemiology field [96], said that “more data are required” [2]. Subsequently, far larger MR studies with much greater statistical power failed to support their preliminary finding. MSR’s attempt to enrol interactions between alcohol intake and genetic polymorphism in cholesteryl ester transfer protein (CETP) in their formulation of an “alcohol->HDL-C-> reduced risk of CHD” causal pathway [97] has likewise failed to survive the test of time. Ironically, adequately powered and designed studies of CETP genetic variation have simultaneously established the non-causal nature of the circulating HDL-C -> CHD association and have demonstrated that any effect of CETP inhibition on CHD is due to its (small) Apo B and LDL cholesterol lowering—rather than its substantial HDL-C elevating—effect [98, 99].

**Fig. 5:**
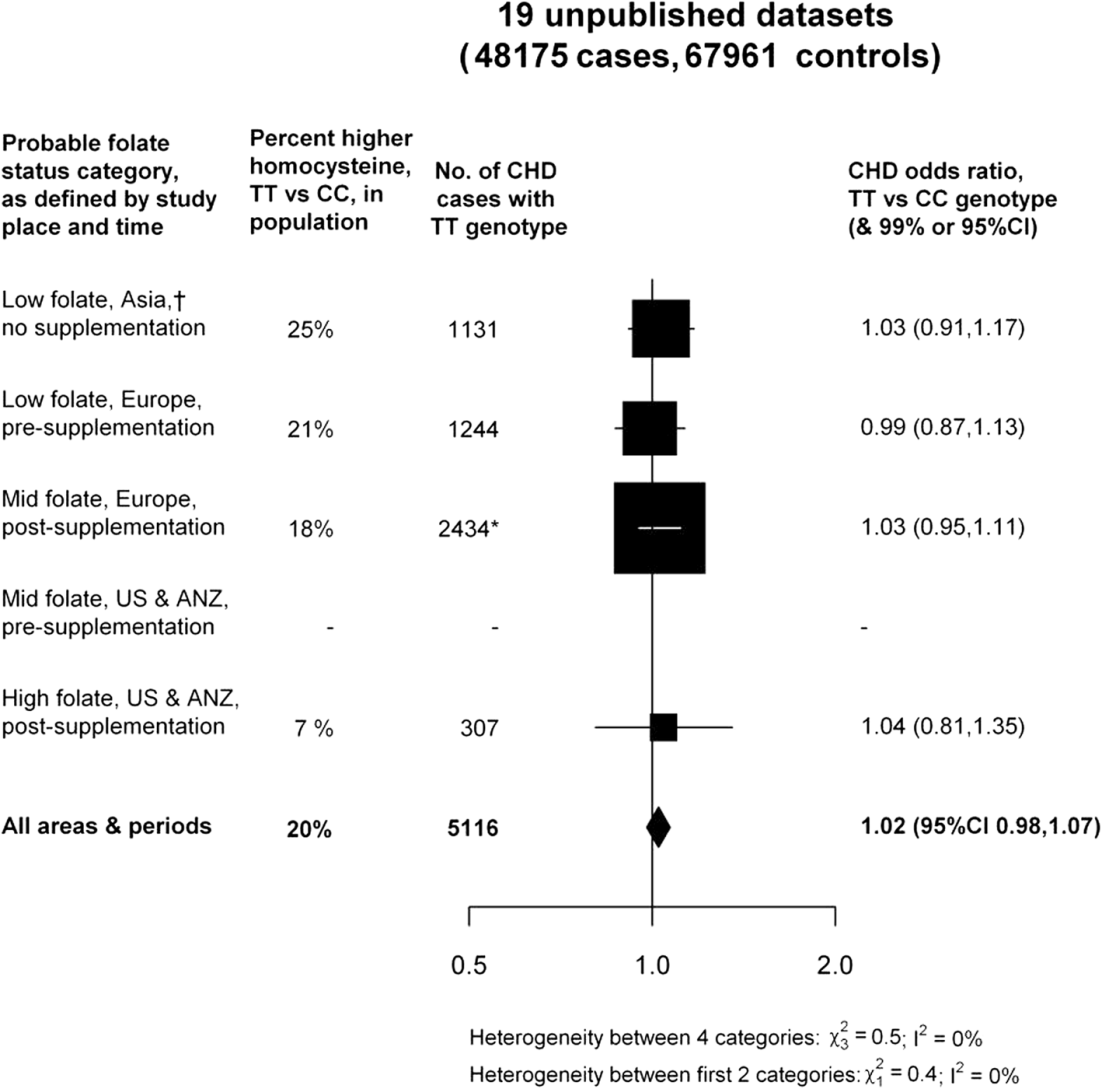
CHD odds ratio (TT versus CC MTHFR C677T genotype) in each probable folate status category, from a meta-analyses of 19 unpublished datasets. *US *United States, *ANZ *Australia, New Zealand) [92]

A similar story of disillusionment is seen with respect to MTHFR (genetic variation in which has been used to suggest involvement of the folate-homocysteine pathway) and CHD. Rimm gave a balanced presentation of MR in a review of folate and vascular disease [100], concluding that “chief among [the limitations of MR] is the need for very large sample sizes” [100]. After these very large sample size MR studies came along, which together with RCTs [93] ruled out a meaningful effect of folate, MSR’s interpretation changed to one which—taken to its logical conclusion—acknowledges that their earlier naïve observational studies produced spurious findings. In our first formulation of MR we used the folate-CHD link as an example of where MR provided supportive evidence [2]. We continued to empirically interrogate this issue as more data accrued and concluded that publication bias was likely responsible for the suggestion that folate was protective in the initial studies [91]. We agree with MSR that selective publication is problematic for MR [1], as it is for conventional epidemiology. Indeed, in the case of MTHFR and CHD researchers have put in the effort to expose and correct it. In an international effort that interrogated our suggestion of publication bias in MTHFR studies [91], Clarke et al. amassed data from unpublished studies—clearly not affected by publication bias—and showed that MTHFR was unrelated to CHD in all populations, including those with low prevailing folate levels [92] (Fig. 5). They triangulated [20, 101] these MR findings with an updated meta-analysis of RCTs of folate supplementation to conclude that together these provided powerful evidence against the claims of substantial effects based on MSR’s and others’ observational studies [92]. We invite readers of the *European Journal of Epidemiology* to consult this paper (and its detailed supplementary material [92]) and contrast it with how MSR have accounted for their clearly erroneous observational epidemiological findings, in which selective publication likely played a role [102, 103].

There is one obvious solution to selective publication, which is making research data accessible. MR studies are increasingly carried out using data that are available to the entire scientific community. This means other investigators can attempt to replicate analyses and can investigate why misleading findings have been generated and published. Such a process led to retraction of an MR paper in which analytical errors were made [36, 104]. The data MSR use are generally not made available; if others could investigate the reasons behind why their methods and publication choices [102, 103] have led to misleading results on many issues this would greatly advance epidemiological rigor [105].

We propose that epidemiological evidence be considered within a triangulation of evidence framework [20, 101]. In such, findings from different study types are evaluated—all of which may be biased—but selected on the grounds that biases across studies are likely to be orthogonal. An example of triangulation of MR findings with those from a meta-analysis of results from RCTs is provided by the above discussion of  folate and CHD [92], and a general framework for triangulation within epidemiology has been proposed [20]. RCTs, of course, provide particularly compelling evidence and examples of where naïve observational studies, MR and RCTs have all been carried out are informative. For example, MSR’s conventional observational studies suggested Vitamin D generates cardiovascular benefits [17]; emerging in parallel, MR and RCTs have consistently suggested this is spurious [19, 106]. Similarly MSR promoted selenium as protective of prostate cancer [16, 107]. RCT [18] and MR evidence [108] suggest it is not. As we have seen above, MSR have been enthusiastic proponents of the CHD-lowering effects of circulating HDL-C; many RCTs and MR studies establish this is spurious. Even RCTs can be biased, and MR studies certainly can, but the potentially orthogonal nature of these biases mean the combined evidence is robust. For example, it could be argued that ~ 5 years exposure in the RCTs is inadequate to produce benefit, but MR studies provide evidence on the effect of life-long differences. Whilst MSR’s epidemiological investigations have provided a very poor basis for planning trials, we consider that funders, beneficiaries (including the public who desire protection from disease without subjection to, at best, useless long term intervention) and clinician-scientists will include MR in their evaluation of which potential interventions should undergo large-scale randomized evaluation.

MSR conclude that we should “get back to the hard problem of conducting high-quality, reproducible epidemiology” [1]. We agree. A first step in improving epidemiology would be to revisit occasions when observational epidemiology has produced highly consequential but misleading findings. After the appearance of their studies suggesting a few years of vitamin E supplement use would substantially reduce CHD risk, the use of supplements containing vitamin E increased substantially [109], and has taken a long time to fall [110], with at best no benefit and potential harm. Surely, both circumspection and exploring how to best conduct high-quality reproducible epidemiology would involve re-examining such situations to uncover why the findings were so misleading, whilst allowing other investigators access to the data for independent investigation? Going forward, rather than focus on nomenclature, let us move to a situation in which triangulation of findings becomes the norm in epidemiology, and methods are considered on the basis of what they have to add to a reliable evaluation of each particular question.
